# A comprehensive immunohistochemical analysis of 26 markers in 250 cases of serous ovarian tumors

**DOI:** 10.1186/s13000-023-01317-9

**Published:** 2023-02-28

**Authors:** Kristýna Němejcová, Adam Šafanda, Michaela Kendall Bártů, Romana Michálková, Jana Drozenová, Pavel Fabian, Jitka Hausnerová, Jan Laco, Radoslav Matěj, Gábor Méhes, Petr Škapa, Ivana Stružinská, Pavel Dundr

**Affiliations:** 1 Institute of Pathology, First Faculty of Medicine, Charles University and General University Hospital in Prague, 12800 Prague, Czech Republic; 2 Department of Pathology, Charles University, 3rd Faculty of Medicine, University Hospital Královské Vinohrady, 10034 Prague, Czech Republic; 3 Department of Oncological Pathology, Masaryk Memorial Cancer Institute, Brno, Czech Republic; 4 Department of Pathology, University Hospital Brno and Medical Faculty, Masaryk University, Brno, Czech Republic; 5 The Fingerland Department of Pathology, Charles University Faculty of Medicine in Hradec Králové and University Hospital Hradec Králové, Hradec Králové, Czech Republic; 6 Department of Pathology and Molecular Medicine, Third Faculty of Medicine, Charles University, Thomayer University Hospital, Prague, Czech Republic; 7 Department of Pathology, Faculty of Medicine, University of Debrecen, 4032 Debrecen, Hungary; 8 Department of Pathology and Molecular Medicine, Second Faculty of Medicine, Charles University and Motol University Hospital, Prague, Czech Republic

**Keywords:** Ovarian tumors, Tubo-ovarian tumors, High grade serous carcinoma, Low grade serous carcinoma, Immunohistochemistry

## Abstract

**Background:**

We examined a large cohort of serous tubo-ovarian tumors with 26 immunohistochemical markers, with the aim to assess their value for differential diagnosis and prognosis.

**Methods:**

Immunohistochemical analyses with 26 immunomarkers were performed on 250 primary tubo-ovarian tumors including 114 high grade serous carcinomas (HGSC), 97 low grade serous carcinomas (LGSC), and 39 serous borderline tumors (micropapillary variant, mSBT). The associations of overall positivity with clinicopathological characteristics were evaluated using the chi-squared test or Fisher’s Exact test.

**Results:**

We found significantly different expression of p53, p16, ER, PR, PTEN, PAX2, Mammaglobin, RB1, Cyclin E1, stathmin, LMP2, L1CAM, CD44, and Ki67 in HGSCs compared to LGSCs. No significant differences were found between LGSC and mSBT. None of the other included markers (PAX8, ARID1A, HNF1B, Napsin A, CDX2, SATB2, MUC4, BRG1, AMACR, TTF1, BCOR, NTRK) showed any differences between the investigated serous tumors. Regarding the prognosis, only PR and stathmin showed a statistically significant prognostic meaning in LGSCs, with better overall survival (OS) and recurrence-free survival (RFS) in cases positive for PR, and worse outcome (RFS) for stathmin. None of the study markers showed prognostic significance in HGSCs.

**Conclusion:**

We provided an extensive immunohistochemical analysis of serous ovarian/tubo-ovarian tumors. Although we found some differences in the expression of some markers in HGSCs compared to LGSCs, only p53, p16, and Ki67 seem to be useful in real diagnostic practice. We also suggested the best discriminative cut-off for Ki67 (10% of positive tumor cells) for distinguishing HGSC from LGSC. We found prognostic significance of PR and stathmin in LGSCs. Moreover, the high expression of stathmin could also be of predictive value in ovarian carcinomas as target-specific anti-stathmin effectors are potential therapeutic targets.

**Supplementary Information:**

The online version contains supplementary material available at 10.1186/s13000-023-01317-9.

## Background

Serous tumors represent the largest subgroup among all epithelial tubo-ovarian tumors, accounting for about 70% of all cases [1]. They are traditionally divided into benign (cystadenomas, adenofibromas, or surface papillomas), borderline (SBT) (conventional and micropapillary/cribriform subtype, mSBT), and malignant, represented by high grade serous carcinoma (HGSC) and low grade serous carcinoma (LGSC). LGSC and HGSC are two distinct tumor types which have different origin, pathogenesis, morphology, molecular characteristics, and prognosis [2].

Diagnosis of serous benign tumors and SBTs usually does not pose a problem. In comparison to the conventional SBT types, micropapillary subtypes have a higher frequency of bilaterality, surface involvement, and extraovarian implants [3].

LGSCs make up approximately 5–7% of all ovarian carcinomas. They mostly present a decade earlier than HGSCs, have lower response to conventional chemotherapy, and have a better clinical outcome in the early stages compared to HGSC [2, 4]. However, the long-term prognosis is poor in the advanced stages [2]. LGSCs are characterised by mild to moderate nuclear atypia (less than a threefold variation in nuclear size) and a variety of the invasive growth pattern, which distinguishes them from SBTs [2].

HGSC are the most common type of epithelial tubo-ovarian carcinoma, accounting for about 70% of all cases, and for the majority of epithelial tubo-ovarian cancer deaths [5]. These tumors typically present in the advanced stages as a large adnexal mass with peritoneal involvement [6]. HGSCs typically exhibit marked cytological atypia often with markedly atypical nuclei (more than threefold variation in nuclear size), and high mitotic activity of > 12 mitoses/10HPF (equating to > 5 mitoses/mm^2^ for HPF diameter of 0.55 mm) [2].

The differentiation of LGSC from HGSC is based mainly on differences in the morphological features, and in difficult cases surrogate immunomarkers such as p53, p16, and Ki67 can be used. Regarding the precise immunohistochemical characterization, so far most studies have been focused on HGSC, while LGSC (due to their rarity) were much less explored as most studies include only a small number of LGSC cases [7–11]. However, some immunohistochemical markers have not been examined in serous tumors at all.

We investigated a large cohort of serous tubo-ovarian tumors including HGSC, LGSC, and mSBT with a panel of 26 antibodies with a view to provide broad immunohistochemical characterization, to assess not only the value of these markers in the differential diagnosis, but also their prognostic significance.

## Methods

### Samples

The archives of the pathology departments of the authors were searched for cases diagnosed as primary tubo-ovarian HGSC, LGSC, and mSBT. The diagnosis of HGSC and LGSC was based on the aforementioned criteria based on nuclear atypia (threefold variation in nuclear size) and the mitotic rate (cut-off 12 mitoses/10HPF, i.e. > 5 mitoses/mm2). All HGSC cases and all but two cases of LGSC/mSBT (with typical morphology and psammoma bodies) were WT1 positive. In total, 250 cases were selected for immunohistochemical analysis, all of which were reviewed by two gynecological pathologists (PD and KN). The sample set included 114 HGSC, 97 LGSC, and 39 SBT (only micropapillary variant), which for the most part represents a dataset of tubo-ovarian tumors already used in our previous study [12]. The clinicopathological and survival characteristics of the 250 patients are summarized in Table 1.

**Table 1:** Clinicopathological and survival characteristics of the dataset of 250 serous ovarian tumors

Characteristics	HGSC, n (%) *n* = 114	LGSC, n (%) *n* = 97	mSBT, n (%) *n* = 39
**Age at diagnosis (years)**			
Median (range)	60 (36–81)	55 (19–83)	51 (25–85)
Mean ± SD	60 ± 10.1	52 ± 14.6	51 ± 14.5
**Follow up (months)**			
Median (range)	38 (0–251)	45 (0–320)	56 (5–189)
Mean ± SD	43 ± 33.1	56 ± 55.9	53 ± 43.3
**FIGO**			
I	9 (8%)	14 (14%)	11 (28%)
II	7 (6%)	5 (5%)	0 (0%)
III	68 (60%)	51 (53%)	16 (41%)
IV	28 (24%)	2 (2%)	0 (0%)
NA	2 (2%)	25 (26%)	12 (31%)
**T-stage**			
T1	13 (11%)	14 (14%)	11 (28%)
T2	13 (11%)	5 (5%)	0 (0%)
T3	85 (75%)	52 (54%)	16 (41%)
Tx/NA	3 (3%)	26 (27%)	12 (31%)
**N-stage**			
N0	29 (25%)	18 (18%)	10 (26%)
N1	44 (39%)	20 (21%)	2 (5%)
Nx/NA	41 (36%)	59 (61%)	27 (69%)
**M-stage**			
M0	69 (61%)	68 (70%)	25 (64%)
M1	28 (24%)	2 (2%)	0 (0%)
Mx/NA	17 (15%)	27 (28%)	14 (36%)
**Survival status**			
NED	28 (24%)	36 (37%)	17 (44%)
AWD	61 (54%)	25 (26%)	5 (13%)
DOD	24 (21%)	12 (12%)	2 (5%)
DOC	0 (0%)	8 (8%)	1 (3%)
NA	1 (1%)	16 (16%)	14 (36%)
**Local recurrence**			
No	53 (47%)	54 (56%)	20 (51%)
Yes	57 (50%)	24 (25%)	5 (13%)
NA	4 (3%)	19 (20%)	14 (36%)
**Distant metastasis**			
No	71 (62%)	62 (64%)	25 (64%)
Yes	39 (34%)	16 (16%)	0 (0%)
NA	4 (4%)	19 (20%)	14 (36%)

Percentages are rounded up/down. *HGSC* High grade serous carcinoma, *LGSC* Low grade serous carcinoma, *mSBT* Micropapillary serous borderline tumor, *NED* No evidence of disease, *AWD* Alive with disease, *DOD* Death of diagnosis, *DOC* Death of other cause, *SD* Standard deviation, *NA* Data not available

### Immunohistochemical analysis

The immunohistochemical analysis was performed using tissue microarrays (TMAs) similarly as described in our previous work [12]. The list of antibodies, their manufacturers, clones, and dilutions are summarized in Supplementary table S1.

The expression of all markers was double-blindly evaluated by two pathologists.

Cases were classified based on the overall percentage of positive cells as negative (entirely negative or < 5% of positive tumor cells) or positive (≥ 5% positive tumor cells) with the exception of p53, p16, and Ki67. The p53 protein expression was assessed as “wild-type” or “aberrant type”. The “aberrant-type” staining was defined as diffuse intense nuclear positivity of > 80% of epithelial cells, cytoplasmic p53 positivity, or complete absence of staining with positive internal control in the form of the “wild-type” staining of variable extent and intensity [10, 13]. The expression of p16 was regarded as block positive (diffuse staining of tumor cells in the nuclear and/or cytoplasmic compartment), or negative (focal/patchy or absent staining). Ki67 was assessed as a continuous variable based on the proportion of positive tumor cells (0–100%). It was counted manually in 250 tumor cells in hot-spots, or in randomly selected fields in cases of homogenous expression [14]. For PTEN, ARID1A, INI1, and BRG1 the loss of expression in tumor cells with retained staining in stromal cells was evaluated.

### Statistical analysis

Group comparisons were performed for continuous (percentage of overall positivity) and categorical (positive vs. negative) variables using the one-way ANOVA and Pearson chi-squared test, or the Fisher Exact test.

Survival curves were constructed by the Kaplan–Meier method and statistically compared by means of the log rank test. Negative versus positive cases were compared for each marker with a sufficient sample size in both categories. Time-to-event analyses were focused on four outcomes: overall survival (OS: the period from the date of diagnosis to the date of recorded death), recurrence-free survival (RFS: the period from the date of curative surgery to the time of recurrence or death), local recurrence-free survival (LFS: the period from the primary diagnosis until the first local recurrence) and distant metastasis-free survival (MFS: the period from the primary diagnosis until the first distant metastasis diagnosis). The follow-up data was available for 213 cases (110 HGSC, 78 LGSC, 25 SBT). The median follow-up in the full cohort was 42 months (range: 0–320, Q1-Q3: 22–26). The longest follow-up was observed in the subset of mSBT (median = 56 months), the shortest in HGSC (median = 37.5 months), but there was no statistically significant difference between the groups (*p* = 0.405). Among the 213 patients, 47 patients died (22%), 38 of them due to diagnosis (81%). Death from diagnosis was more frequently seen in HGSC (22%) compared to the LGSC/mSBT group (14%), but this trend was not significant (*p* = 0.117).

A receiver-operating characteristic curve (ROC) and the optimal cut-off values were established using the library “pROC” and “cutpointr” implemented in R. *P*-value of < 0.05 was considered as significant. All statistical analyses were performed using the program R, version 4.1.1 (https://cran.r-project.org/).

## Results

**Fig. 1:**
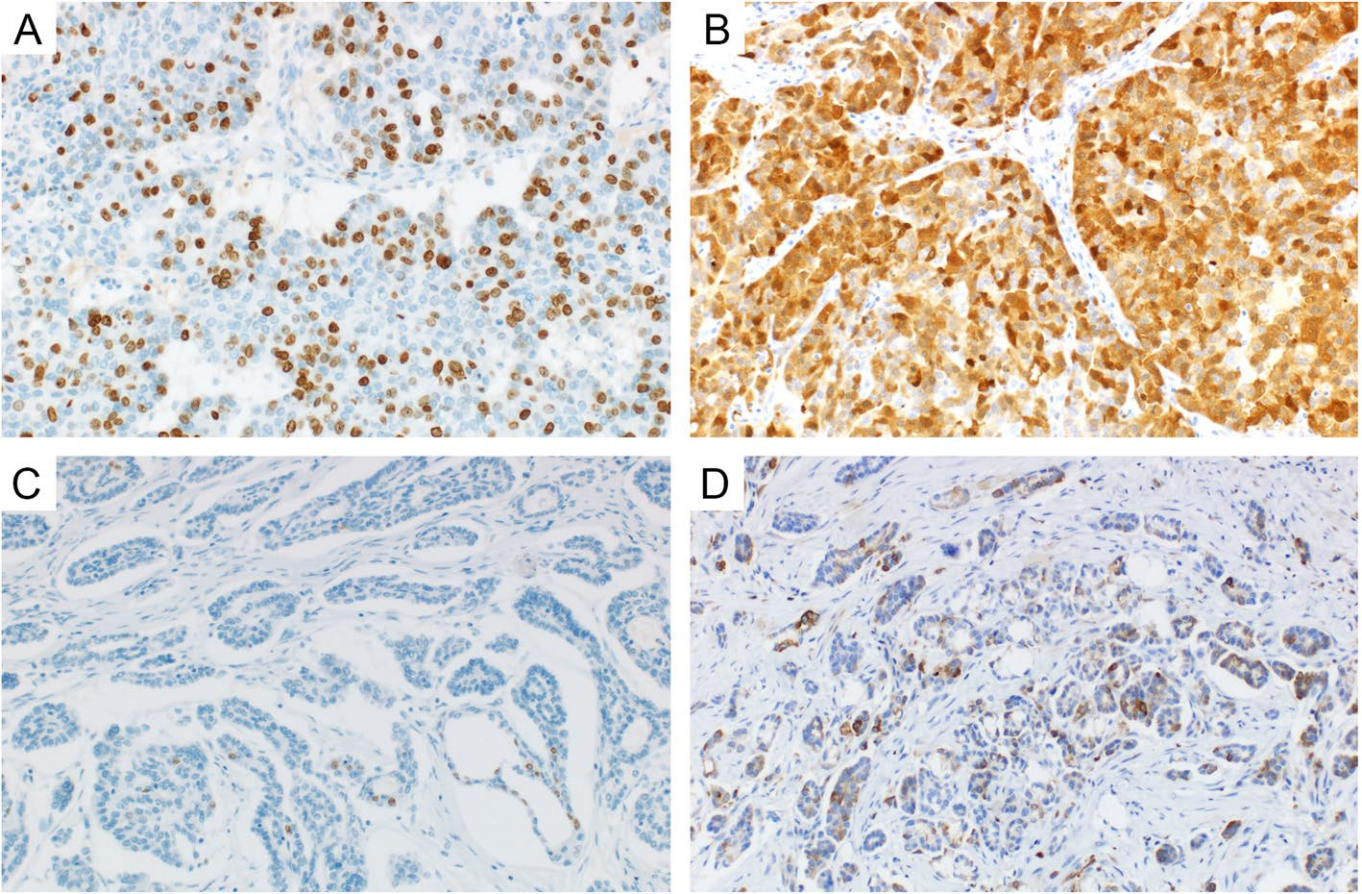
Representative IHC staining of Ki67 and stathmin in HGSC and LGSC cases. **A** Expression of Ki67 in HGSC (200x), **B** Expression of stathmin in HGSC (200x), **C** Expression of Ki67 in LGSC (200x), D) Expression of stathmin in LGSC (200x). HGSC = high grade serous carcinoma, LGSC = low grade serous carcinoma

The results of immunohistochemical analyses in relation to the individual tumor types are summarized in Tables 2 and 3 (see also Fig. 1).

**Table 2:** Overview of overall positivity (%) of 26 markers and differences between tumor types

Marker	HGSC (*n* = 114)	LGSC (*n* = 97)	mSBT (*n* = 39)	HGSC vs. (LGSC + mSBT)* p*-value	HGSC vs. LGSC *p*-value	LGSC vs. mSBT *p-*value
ER				**< 0.001**	** < 0.001**	0.998
n	114	94	39			
range (mean/median)	0–100 (72/90)	0–100 (91.1/98)	68–100 (95/98)			
PR				** < 0.001**	**0.004**	**0.003**
n	114	97	39			
range (mean/median)	0–100 (8.7/0)	0–100 (19.1/5)	0–90 (29.8/20)			
PAX8				**< 0.001**	**< 0.001**	0.408
n	114	96	39			
range (mean/median)	0–100 (96.5/100)	2–100 (81.5/90)	45–100 (86.2/92)			
ARID1A				** < 0.001**	**< 0.001**	0.752
n	114	95	38			
range (mean/median)	0–100 (88.5/97.5)	0–100 (57.8/60)	1–100 (57.6/65)			
PTEN				**< 0.001**	**< 0.001**	0.953
n	114	96	38			
range (mean/median)	0–100 (54.1/62.5)	0–100 (76.9/85)	20–100 (78.1/83.5)			
HNF1B				0.878	0.800	0.304
n	114	93	34			
range (mean/median)	0–98 (14.2/0)	0–100 (15.4/0)	0–100 (21.5/0)			
Napsin A				NULL	NULL	NULL
n	114	96	39			
range (mean/median)	0	0	0			
CDX2				0.706	0.757	0.927
n	114	97	39			
range (mean/median)	0–95 (1.7/0)	0–10 (0.1/0)	0			
NTRK				0.906	0.914	NULL
n	114	96	39			
range (mean/median)	0–21 (0.2/0)	0	0			
MUC4				0.463	0.525	0.927
n	114	97	39			
range (mean/median)	0–30 (0.6/0)	0–7 (0.1/0)	0			
BRG1				**< 0.001**	**< 0.001**	0.893
n	114	97	39			
range (mean/median)	21–100 (89.9/95)	0–100 (99/100)	95–100 (99.9/100)			
PAX2				**< 0.001**	**< 0.001**	0.171
n	114	95	37			
range (mean/median)	0–98 (15.8/0)	0–100 (41.5/22)	0–100 (51.8/64)			
Mammaglobin				**0.002**	**0.001**	**0.034**
n	114	96	37			
range (mean/median)	0–95 (4.7/0)	0–75 (0.9/0)	0–22 (1.1/0)			
SATB2				0.318	0.424	0.780
n	114	97	39			
range (mean/median)	0–31 (0.9/0)	0–40 (0.5/0)	0			
RB1				**0.002**	**0.007**	0.994
n	113	92	39			
range (mean/median)	0–100 (68.6/90)	0–100 (64.1/74)	20–97 (67.4/67)			
AMACR				0.993	0.822	0.544
n	114	96	39			
range (mean/median)	0–75 (2.4/0)	0–80 (2.6/0)	0–5 (0.3/0)			
TTF1				0.905	0.914	NULL
n	114	97	39			
range (mean/median)	0–5 (0.1/0)	0	0			
Cyclin E1				**< 0.001**	**< 0.001**	0.592
n	114	93	37			
range (mean/median)	0–100 (67.2/79)	0–90 (23.9/17)	0–75 (21.2/15)			
Statmin				**< 0.001**	** < 0.001**	0.404
n	114	94	38			
range (mean/median)	0–100 (70.2/80)	0–98 (23.9/12)	1–70 (15.4/8.5)			
BCOR				0.278	0.273	0.817
n	114	97	39			
range (mean/median)	0–4 (0.2/0)	0	0–4 (0.1/0)			
L1CAM				**0.046**	0.134	0.484
n	114	95	37			
range (mean/median)	0–100 (12.0/0)	0–90 (7.6/0)	0–60 (2.4/0)			
LMP2				**< 0.001**	** < 0.001**	0.264
n	114	95	38			
range (mean/median)	0–100 (72.6/95)	45–100 (95.3/100)	60–100 (98.3/100)			
CD44				**< 0.001**	**< 0.001**	0.753
n	114	93	37			
range (mean/median)	0–100 (6.9/0)	0–12 (0.5/0)	0–6 (0.3/0)			
Ki67				** < 0.001**	**< 0.001**	0.948
n	114	91	37			
range (mean/median)	0–95 (45.6/44)	0–29 (3.0/1)	0–5 (2.0/2)			

*p*-values are based on Mann–Whitney U test, NULL = values could not be generated, significant differences are indicated in bold font

*HGSC* High grade serous carcinoma, *LGSC* Low grade serous carcinoma, *mSBT* Micropapillary serous borderline tumor, *NULL* values could not be generated

**Table 3:** Overview of the expression of 26 markers based on categorical scoring: positive (≥ 5% positive tumor cells) vs. negative (< 5% positive tumor cells) cases

Marker	HGSC (*n* = 114)	LGSC (*n* = 97)	mSBT (*n* = 39)	HGSC vs. (LGSC + mSBT)* p*-value	HGSC vs. LGSC *p*-value	LGSC vs. mSBT*p*-value
ER				**< 0.001**	**0.004**	1.000
n positive	101 (89%)	93 (99%)	39 (100%)			
n negative	13 (11%)	1 (1%)	0 (0%)			
PR				**< 0.001**	**0.005**	**< 0.001**
n positive	36 (32%)	49 (51%)	32 (82%)			
n negative	78 (68%)	48 (49%)	7 (18%)			
PAX8				1.000	1.000	1.000
n positive	113 (99%)	95 (99%)	39 (100%)			
n negative	1 (1%)	1 (1%)	0 (0%)			
ARID1A				0.068	0.082	1.000
n positive	112 (98%)	88 (93%)	36 (95%)			
n negative	2 (2%)	7 (7%)	2 (5%)			
PTEN				**< 0.001**	**< 0.001**	1.000
n positive	96 (84%)	95 (99%)	38 (100%)			
n negative	18 (16%)	1 (1%)	0 (0%)			
HNF1B				0.801	0.538	0.294
n positive	34 (30%)	24 (26%)	12 (35%)			
n negative	80 (70%)	69 (74%)	22 (65%)			
Napsin A				NULL	NULL	NULL
n positive	0 (0%)	0 (0%)	0 (0%)			
n negative	114 (100%)	96 (100%)	39 (100%)			
CDX2				0.334	0.626	1.000
n positive	3 (3%)	1 (1%)	0 (0%)			
n negative	111 (97%)	96 (99%)	39 (100%)			
NTRK				0.458	1.000	NULL
n positive	1 (1%)	0 (0%)	0 (0%)			
n negative	113 (99%)	96 (100%)	39 (100%)			
MUC4				0.181	0.377	1.000
n positive	4 (4%)	1 (1%)	0 (0%)			
n negative	110 (96%)	96 (99%)	39 (100%)			
BRG1				1.000	0.459	1.000
n positive	114 (100%)	96 (99%)	39 (100%)			
n negative	0 (0%)	1 (1%)	0 (0%)			
PAX2				**< 0.001**	**< 0.001**	0.118
n positive	47 (41%)	64 (67%)	30 (81%)			
n negative	67 (59%)	31 (33%)	7 (19%)			
Mammaglobin				**0.009**	**0.002**	0.050
n positive	16 (14%)	2 (2%)	4 (11%)			
n negative	98 (86%)	94 (98%)	33 (89%)			
SATB2				0.475	0.728	0.557
n positive	5 (4%)	3 (3%)	0 (0%)			
n negative	109 (96%)	94 (97%)	39 (100%)			
RB1				** < 0.001**	**0.002**	1.000
n positive	97 (86%)	90 (98%)	39 (100%)			
n negative	16 (14%)	2 (2%)	0 (0%)			
AMACR				0.588	0.461	0.508
n positive	8 (7%)	10 (10%)	2 (5%)			
n negative	106 (93%)	86 (90%)	37 (95%)			
TTF1				0.456	1.000	NULL
n positive	1 (1%)	0 (0%)	0 (0%)			
n negative	113 (99%)	97 (100%)	39 (100%)			
Cyclin E1				**< 0.001**	** < 0.001**	0.743
n positive	108 (95%)	68 (73%)	26 (70%)			
n negative	6 (5%)	25 (27%)	11 (30%)			
Statmin				**< 0.001**	** < 0.001**	0.431
n positive	110 (96%)	66 (70%)	24 (63%)			
n negative	4 (4%)	28 (30%)	14 (37%)			
BCOR				NULL	NULL	NULL
n positive	0 (0%)	0 (0%)	0 (0%)			
n negative	114 (100%)	97 (100%)	39 (100%)			
L1CAM				**0.002**	**0.045**	**0.037**
n positive	39 (34%)	20 (21%)	2 (5%)			
n negative	75 (66%)	75 (79%)	35 (95%)			
LMP2				**0.002**	**0.009**	NULL
n positive	106 (93%)	95 (100%)	38 (100%)			
n negative	8 (7%)	0 (0%)	0 (0%)			
CD44				** < 0.001**	** < 0.001**	1.000
n positive	30 (26%)	3 (3%)	1 (3%)			
n negative	84 (74%)	90 (97%)	36 (97%)			
Ki67				**< 0.001**	**< 0.001**	0.573
n positive	109 (96%)	16 (18%)	5 (14%)			
n negative	5 (4%)	75 (82%)	32 (86%)			
p16^a^				**< 0.001**	**< 0.001**	NULL
n positive	77 (68%)	0 (0%)	0 (0%)			
n negative	37 (32%)	96 (100%)	38 (100%)			
p53^a^				**< 0.001**	**< 0.001**	NULL
n positive	98 (91%)	0 (0%)	0 (0%)			
n negative	10 (9%)	96 (100%)	38 (100%)			

^a^p53 protein expression was assessed as negative (“wild-type”) or positive (“aberrant type”). p16 was assessed as positive (block positivity) or negative (only focal or absent staining, see Methods section—Immunohistochemical analysis)

*p*-values are based on Pearson chi-squared or Fisher Exact test, significant differences are indicated bold font

*HGSC* High grade serous carcinoma, *LGSC* Low grade serous carcinoma, *mSBT* Micropapillary serous borderline tumor, *NULL* Values could not be generated

Briefly, aberrant staining pattern of p53 was found in 91% (97/114; 67% overexpression, 24% null expression) of HGSC. No cases of LGSC showed aberrant p53 expression. Expression of p16 was diffusely positive in 68% (77/114) of HGSC, while no LGSC (or SBTs) showed diffuse expression.

**Fig. 2:**
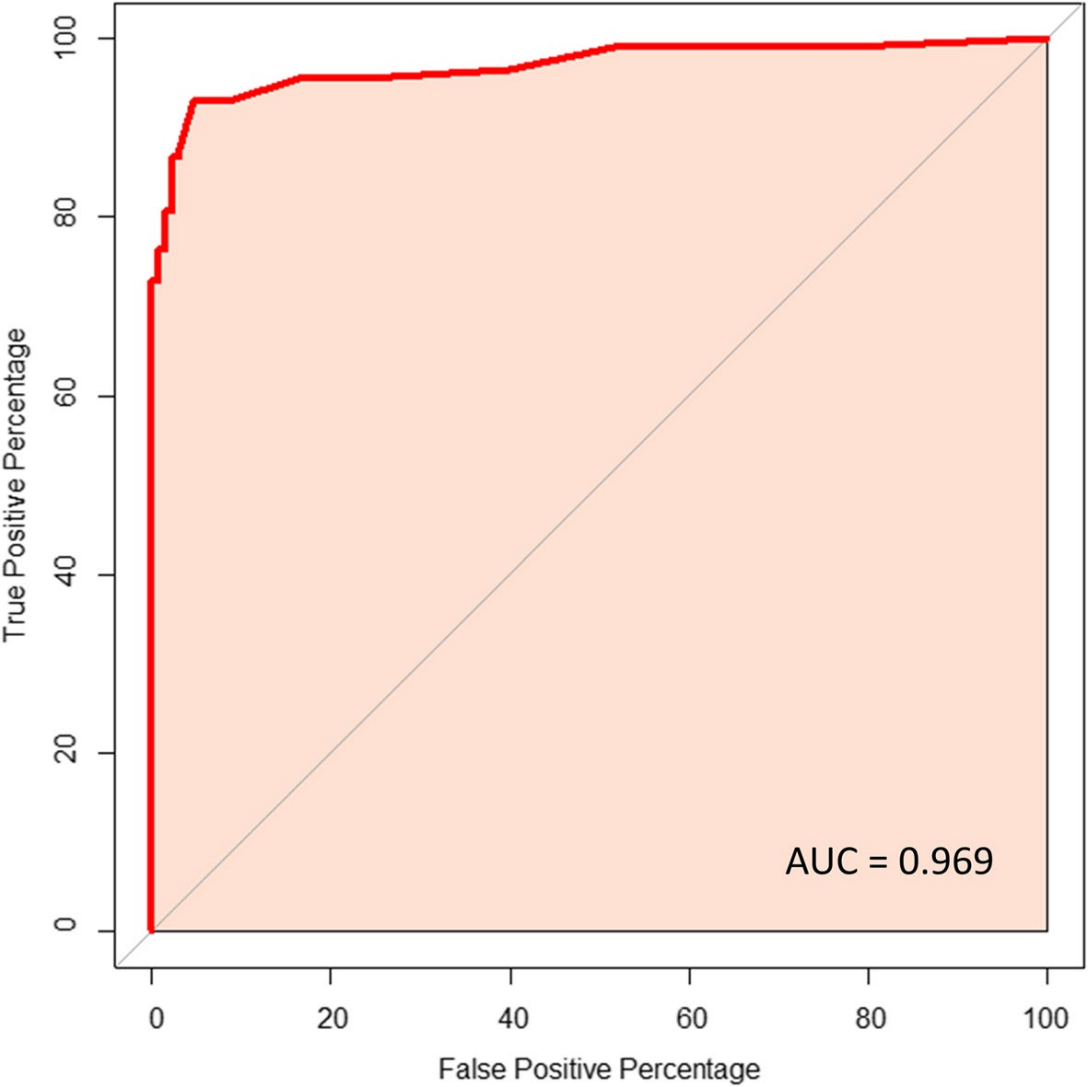
The Receiver Operating Characteristic (ROC) curve for Ki67 and the applicability of this marker in the differential diagnosis between HGSC and LGSC/mSBT

We found significantly different expression (number of positive vs negative cases) of p53, p16, ER, PR, PTEN, PAX2, Mammaglobin, RB1, Cyclin E1, stathmin, LMP2, L1CAM, CD44, and Ki67 in HGSC compared to LGSC (Table 3). Similar results were also obtained when the expression was analysed as a continuous variable (0–100%) (Table 2). There were also significant differences in the expression of PAX8, ARID1A, and BRG1 between HGSC and LGSC. For these markers we performed analyses of the optimal cut-point for distinguishing between HGSC and LGSC (Supplementary table S2). We also analyzed the expression of markers that seem to be useful for differential diagnosis (Ki67, Cyclin E1, and PAX2). Based on our data, the ideal cut-off for distinguishing between HGSC and LGSC was 10% for Ki67, ~ 90% for ARID1A, and ~ 50% for Cyclin E1. However, in terms of real practice the only usable cut-off is the 10% for Ki67, with the sensitivity = 0.929 and specificity = 0.953 (Fig. 2). Other markers did not show sufficient sensitivity and/or specificity (Supplementary table S2).

Of all the markers, only PR showed differences in expression between LGSC and SBT, with 51% positivity in LGSC (mean/median of % positivity = 19.1/5), and 82% positivity in SBT (mean/median of % positivity = 29.8/20).

### Survival analyses

Survival analyses were performed separately for HGSC and LGSC/mSBT cases with known follow-up, and only for markers with a sufficient number of events of interest in both the positive and negative categories.

**Fig. 3:**
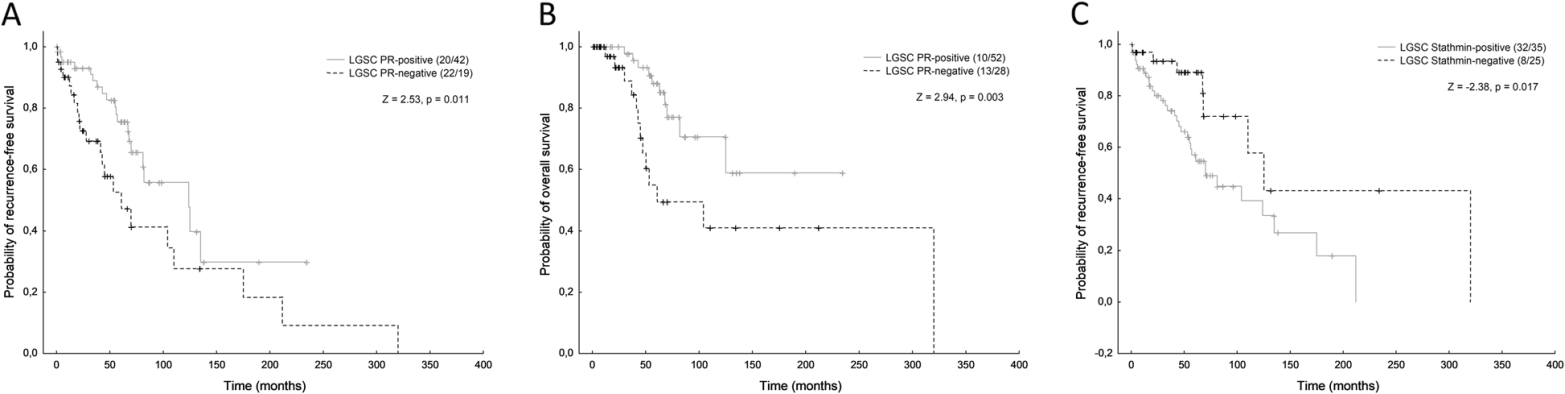
Survival analyses showing better prognosis for PR positive or stathmin negative LGSCs. Kaplan–Meier curves for **A**) recurence-free survival and **B**) overall survival in relation to the expression of PR and **C**) recurence-free survival in relation to the expression of stathmin in a subset of LGSC/mSBT. The *p*-values are based on log rank test, the number of complete/censored cases is stated in the parentheses

In HGSC no difference in survival in relation to the expression of examined markers was detected. In LGSC, only PR and stathmin showed any statistically significant difference in prognosis, with a better outcome for the PR positive cases and a worse outcome for the stathmin positive cases (Fig. 3). PR-positive cases showed better overall survival and recurrence-free survival (*p* = 0.003 for OS, *p* = 0.011 for RFS), while for stathmin the trend was opposite and better RFS was observed in negative cases (*p* = 0.017, Fig. 3).

## Discussion

We provide an extensive immunohistochemical analysis of serous ovarian/tubo-ovarian carcinomas and mSBTs with 26 immunomarkers, with the aim to better characterize immunohistochemical profile of these tumors, in which we found differences in expression potentially useful in differential diagnosis. Some of the markers such as p53, p16, and Ki67 have been described in literature (mostly in HGSC), while others have not been investigated at all, or only in a small number of studies [10, 14–16]. When considering LGSC, as a much rare tumor type, large studies focusing primarily on the immunohistochemical profile of LGSCs are missing. Most published studies were not primarily focused on this tumor type, and LGSCs were often mentioned together with other tumor types with only a limited number of cases.

We also focused on the differential diagnosis of serous tubo-ovarian tumors, which is mostly based on their morphological features. Difficult cases usually require immunohistochemistry, especially p53, p16, and Ki67. The loss of RB1 expression is also more common in HGSC and could be of use [7]. However, none of other investigated markers in our study proved to be useful in the differential diagnosis of tubo-ovarian serous tumors. Although we found statistically significant differences in the expression in ER, PR, PTEN, PAX2, mammaglobin, cyclin E1, stathmin, L1CAM, and CD44, their value for differential diagnosis is rather limited, because the assessment of suitable cut-offs for positivity would be problematic in diagnostic practice. From the above-mentioned markers ER, PR, PTEN, and PAX2 expression was more prevalent in LGSC (98%, 50%, 99%, 66%) compared to HGSC (89%, 32%, 84%, 41%). On the contrary, the expression of CD44, mammaglobin, cyclin E1, stathmin, and L1CAM was more prevalent in HGSC (26%, 14%, 95%, 96%, 34%) than LGSC (3%, 2%, 74%, 71%, 21%). We found no diagnostically useful differences in the immunoprofile of LGSC and mSBT.

We also included other markers mostly used in the differentiation of non-serous ovarian and metastatic tumors, such as MUC4, CDX2, SATB2, HNF1B, napsin A, TTF1, AMACR, and ARID1A which have not yet been thoroughly investigated on a large sample set of serous tumors, and we found positive expression of these markers in only a small number of cases. The only exception was HNF1B with expression reaching up to 30% (30% HGSCs 26% LGSCs) of cases. However, none of the LGSCs showed strong nuclear positivity, and only eight cases of HGSCs showed focal strong positivity in up to 30% tumor cells, in contrast to clear cell carcinomas which are often diffusely strongly positive. No relevant differences were found between LGSC and HGSC.

The literary data concerning p53 expression in serous tubo-ovarian tumors are mostly focused on HGSC, in which the p53 aberrant expression pattern is reported in the range of 89–98% (overexpression pattern in 57–71%, complete absence of staining in 23–32% of HGSC) [10, 15–17]. These results are in concordance with ours. According to the WHO classification, LGSC should show p53 wild type expression; however, some studies described the aberrant expression p53 in quite a wide range of 0–90% of cases [2, 15, 16, 18]. In the study by Sallum et al. 90% (19/21) of LGSCs demonstrated diffuse expression or complete absence of p53, while focal expression (wild type) was found only in 9.5% (2/21) LGSCs [16]. The authors investigated the potential use of combining the expression patterns of p53 and p16 but, as they stated, the morphologic classification showed a better association with survival [16]. In the study by Altman et al. aberrant p53 expression was found in 9% (4/45) LGSCs and in 6% (3/49) SBTs [15]. However, the results of our study showed wild-type expression in all LGSC, which agrees with most other studies [2, 18]. The p53 aberrant expression in tumors morphologically classified as LGSC could reflect the fact that a minority of HGSCs can arise from LGSCs, so this finding could represent high grade transformation [19, 20]. Some cases with overlapping features between LGSC and HGSC, the so called “indeterminate grade serous carcinomas” [21] have also been described. These tumors mostly have the architectural patterns of LGSC, with the presence of areas with high grade nuclear atypia and higher mitotic index mixed with areas of small uniform nuclei that resemble LGSC, and they probably bear similar unfavorable or even worse prognosis than HGSC.

The other corroborative markers used in distinguishing HGCS and LGSC are p16 and Ki67. About 50–80% of HGSCs and up to 6% of LGSCs show diffuse p16 expression [2, 7, 15, 22, 23]. The expression of Ki67 in HGSC is usually higher compared to LGSC. Those studies investigating Ki67 expression in HGSC described a median of 38% to 65% (range 3.6–89%). In our study the was median 44% (range 0–95%) [14, 24–27]. LGSC show usually lower Ki67 expression with a median of 2.5–7% (range 0.28–26%) [25, 28], with our LGSC cases showing a median of 1.5 (range 0–29%). The slight observed differences can be attributed to different methodology. A rather high ki67 index was described in only one study (range 10–40%, mean 19.4%) [27]. However, 16.7% (3/18) of their LGSC cases also showed aberrant p53 expression.

Currently, no precise Ki67 cut-off for distinguishing LGSC from HGSC is universally accepted. Köbel et al. used 13% as a cut-off for high versus low Ki67 labelling index, effectively separating HGSC from LGSC, endometrioid, and clear cell carcinomas [25]. We have focused only on serous tubo-ovarian tumors and the results showed 10% as a statistically relevant cut-off for distinguishing HGSC from LGSC/SBT (sensitivity 0.929, specificity 0.953).

From the evaluated markers, only PR and stathmin showed statistically significant prognostic meaning in our LGSC/mSBT sample set, with a better outcome in PR positive cases (OS and RFS), and a worse outcome in stathmin positive cases (RFS). Most studies found a positive correlation between PR and better survival outcomes in HGSC, but the prognostic role of ER remains ambiguous [29–31]. Matsou et al. found a positive correlation of ER expression and lymphovascular invasion, which was an independent prognostic indicator of poor survival outcomes in HGSC [32]. Chen et al. found a positive association between ER/PR positivity and peritoneal metastases [33]. The results of those studies dealing with outcomes in LGSC seem equivocal. The metanalysis from Shen et al. found ER expression to be associated with improved overall survival in epithelial ovarian cancer [34]. Others found a relationship between better PFS and low PR expression and between longer OS and high ER expression in univariate analysis [35].

The overexpression of stathmin is associated with poor clinico-pathological variables in a lot of malignant tumors [36–40]. In tubo-ovarian carcinomas, attention was mostly focused to HGSC where stathmin expression was analyzed in relation to tumorigenesis and diagnostic utility, and the reported positivity ranges between 84–94% HGSC [9, 41]. In LGSC, the stathmin expression was only briefly mentioned in one study on 26 LGSC cases, but they did not provide the number of positive LGSC cases [42]. Our study is the first one focused on the prognostic impact of stathmin in serous carcinomas on an immunohistochemical level, but we did not confirm any association between the expression and examined clinico-pathological or prognostic parameters.

The stathmin expression could potentially be of use for targeted therapy. A variety of target-specific anti-stathmin effectors were used in *invitro*/in vivo studies on a broad range of tumors; however, these will require further exploration [43]. Regarding the predictive marker NTRK, only one case of HGSC showed weak cytoplasmic expression.

## Conclusion

We provided an extensive immunohistochemical analysis and characterization of serous tumors, especially LGSC, which had not yet been performed. The results of our study showed only a limited value of the examined markers for the differential diagnosis of serous tubo-ovarian epithelial tumors, except for p53, p16, and Ki67. Based on our analysis, we suggested the best discriminative cut-off for Ki67 (10% of positive tumor cells) for distinguishing HGSC from LGSC. Although we found some differences in the expression of some other markers, the practical value of these for differential diagnosis seems to be rather limited. We did not find any useful differences concerning the immunohistochemical expression between LGSC and SBT.

Regarding prognostic meaning, our study showed an association of PR and stathmin with better outcomes (OS, RFS) in the PR positive cases, and worse outcomes (RFS) for stathmin positive LGSC. The expression of stathmin has so far been investigated in only a handful of studies focused on HGSC and the detailed data on LGSC is missing entirely, although it could be of predictive value in tubo-ovarian carcinomas since target-specific anti-stathmin effectors now represent potential therapeutic targets.

## Supplementary Information

**Additional file 1: TableS1. **List of antibodies. **TableS2.** Overview of optimal cut-offs for the selected markers distinguishingbetween HGSC vs. LGSC and LGSC vs. mSBT. Suitable markers are marked in bold,based on sensitivity and specificity. 

## Data Availability

All data generated or analyzed during this study is included in this published article (and its Supplementary information files).
