# Supplementary material for: A comprehensive immunohistochemical analysis of 26 markers in 250 cases of serous ovarian tumors
Source: Diagn Pathol. 2023 Feb 28;18:32. doi: 10.1186/s13000-023-01317-9 (PMC9972686; doi:10.1186/s13000-023-01317-9)
Supplement: Supplementary file 1 — Additional file 1: TableS1. List of antibodies. TableS2. Overview of optimal cut-offs for the selected markers distinguishingbetween HGSC vs. LGSC and LGSC vs. mSBT. Suitable markers are marked in bold,based on sensitivity and specificity. [file 13000_2023_1317_MOESM1_ESM.docx]

Supplementary table S1. List of antibodies.

| **Marker** | **Supplier** | **clone** | **Primary dilution** | **Antigen retrieval** | **Detection** | **Evaluated expression** |
| --- | --- | --- | --- | --- | --- | --- |
| **SATB2** | Cell Marque | EP281 | 1:400 | HIER (pH 9) | EnVision FLEX, Dako | Nuclear |
| **Mammaglobin** | Zytomed | 31A5 | 1:200 | HIER (pH 9) | EnVision FLEX, Dako | Cytoplasmic |
| **NTRK** | Abcam | EPR17341 | 1:100 |  | Optiview Amplification Kit, Ventana | Cytoplasmic and/or nuclear |
| **L1CAM / CD171** | Invitrogen | UJ127 | 1:100 |  | Optiview Amplification Kit, Ventana | Membranous |
| **CD44** | Dako | DF1485 | 1:100 | HIER (pH 9) | EnVision FLEX, Dako | Membranous |
| **MUC4** | Bio SB | EP256 | 1:400 | HIER (pH 6) | EnVision FLEX, Dako | Cytoplasmic |
| **LMP2** | Abcam | polyclonal | 1:1000 |  | UltraView Detection Kit, Ventana | Cytoplasmic and nuclear |
| **CDX2** | Zytomed | EPR2764Y | 1:200 |  | Optiview Amplification Kit, Ventana | Nuclear |
| **TTF1** | BioCare | SPT24 | 1:200 |  | Optiview Amplification Kit, Ventana | Nuclear |
| **BCOR** | Santa Cruz Biotechnology | C-10 | 1:50 |  | Optiview Amplification Kit, Ventana | Nuclear |
| **PAX2** | DCS | EP2325 | 1:100 | HIER (pH 9) | EnVision FLEX, Dako | Nuclear |
| **PAX8** | Cell Marque | polyclonal | 1:50 | HIER (pH 9) | EnVision FLEX, Dako | Nuclear |
| **NapsinA** | Novocastra | IP64 | 1:200 | HIER (pH 9) | EnVision FLEX, Dako | Cytoplasmic |
| **INI1** | Cell Marque | MRQ27 | 1:50 | HIER (pH 9) | EnVision FLEX, Dako | Nuclear |
| **Stathmin** | Bio SB | EP247 | 1:100 | HIER (pH 9) | EnVision FLEX, Dako | Cytoplasmic |
| **Cyclin E1** | Bio SB | EP126 | 1:100 | HIER (pH 9) | EnVision FLEX, Dako | Nuclear |
| **ER** | Zytomed | SP1 | 1:200 |  | Optiview Amplification Kit, Ventana | Nuclear |
| **PR** | Novocastra | 16 | 1:100 |  | Optiview Amplification Kit, Ventana | Nuclear |
| **ARID1** | Santa Cruz Biotechnology | PSG3 | 1:500 | HIER (pH 9) | EnVision FLEX, Dako | Nuclear |
| **BRG1** | Abcam | EPNCIR111A | 1:100 | HIER (pH 9) | EnVision FLEX, Dako | Nuclear |
| **PTEN** | Dako | 6H2.1 | 1:200 | HIER (pH 9) | EnVision FLEX, Dako | Cytoplasmic and nuclear |
| **Ki67** | Dako | MIB-1 | 1:100 |  | UltraView Detection Kit, Ventana | Nuclear |
| **p16** | Diagnostic BioSystems | JC2 | 1:200 |  | Optiview Amplification Kit, Ventana | Cytoplasmic and nuclear |
| **RB1** | Bio Sb | 1F8 | 1:25 | HIER (pH 9) | EnVision FLEX, Dako | Nuclear |
| **AMACR** | Dako | 13H4 | 1:200 | HIER (pH 9) | EnVision FLEX, Dako | Cytoplasmic |
| **p53** | Dako | DO-7 | 1:400 |  | UltraView Detection Kit, Ventana | Cytoplasmic and nuclear |

HIER = heat induced epitope retrieval

Supplementary table S2: Overview of optimal cut-offs for the selected markers distinguishing between HGSC vs. LGSC and LGSC vs. mSBT. Suitable markers are marked in bold, based on sensitivity and specificity.

|  | **HGSC vs. (LGSC+mSBT)** | **HGSC vs. LGSC** | **LGSC vs. mSBT** |
| --- | --- | --- | --- |
| **PAX8** |  |  |  |
| cut-off (% positivity) | 97 | 97 | 72 |
| sensitivity | 0.859 | 0.859 | 0.871 |
| specificity | 0.693 | 0.679 | 0.219 |
| AUC | 0.789 | 0.784 | 0.523 |
| **ARID1A** |  |  |  |
| cut-off (% positivity) | **91** | **91** | 20 |
| sensitivity | **0.746** | **0.746** | 0.947 |
| specificity | **0.842** | **0.821** | 0.168 |
| AUC | **0.829** | **0.821** | 0.482 |
| **BRG1** |  |  |  |
| cut-off (% positivity) | 100 | 100 | 95 |
| sensitivity | 0.985 | 0.989 | 1.000 |
| specificity | 0.640 | 0.640 | 0.010 |
| AUC | 0.811 | 0.812 | 0.493 |
| **Ki67** |  |  |  |
| cut-off (% positivity) | **10** | **10** | 1 |
| sensitivity | **0.929** | **0.929** | 0.837 |
| specificity | **0.953** | **0.934** | 0.257 |
| AUC | **0.969** | **0.966** | 0.496 |
| **Cyclin E1** |  |  |  |
| cut-off (% positivity) | **53** | **47** | 20 |
| sensitivity | **0.719** | **0.763** | 0.484 |
| specificity | **0.900** | **0.849** | 0.622 |
| AUC | **0.847** | **0.842** | 0.530 |
| **PAX2** |  |  |  |
| cut-off (% positivity) | 47 | 40 | 8 |
| sensitivity | 0.477 | 0.473 | 0.783 |
| specificity | 0.877 | 0.859 | 0.400 |
| AUC | 0.706 | 0.690 | 0.577 |

HGSC = high grade serous carcinoma, LGSC = low grade serous carcinoma, mSBT = micropapillary serous borderline tumor; AUC = area under curve
